# Zipline Into a Case of Spontaneous Cerebrospinal Fluid Rhinorrhea

**DOI:** 10.7759/cureus.17277

**Published:** 2021-08-18

**Authors:** Shahan Haseeb, Syed A Bokhari, Muhammad Umer Riaz Gondal, Hadia Wali, Shayan S Ansari

**Affiliations:** 1 Internal Medicine, Shifa International Hospital, Islamabad, PAK; 2 Otolaryngology - Head and Neck Surgery, Shifa International Hospital, Islamabad, PAK

**Keywords:** spontaneous csf rhinorrhea, csf leakage, cerebrospinal fluid (csf), traumatic csf leak, zip line

## Abstract

Spontaneous cerebrospinal fluid (CSF) rhinorrhea is an uncommon phenomenon. One of the complications associated with CSF rhinorrhea is meningitis, which is associated with high mortality. Therefore, the prompt diagnosis of CSF rhinorrhea is essential. We present a case of a patient, who after zip-lining, developed CSF rhinorrhea. She had no history of trauma and none of the conventional comorbidities associated with spontaneous CSF rhinorrhea. She was diagnosed with the help of radiological studies and biochemical tests. Our case is unique as there are no published case reports of spontaneous CSF rhinorrhea occurring after atraumatic zip-lining.

## Introduction

Cerebrospinal fluid (CSF) is a clear fluid that surrounds the brain and spinal cord. CSF rhinorrhea occurs when there is a fistula between the dura and the skull base and discharge of CSF from the nose. CSF rhinorrhea can be divided into traumatic and non-traumatic [1]. The majority of traumatic fistula are accidental (80%), with iatrogenic accounting for around 16%. Spontaneous CSF leak is fairly uncommon, making up around 3-4% of cases [2]. It can occur either due to raised intracranial pressure or variations in CSF pressure [3].

We report a case of a patient, with no history of trauma, who presented with right-sided nasal discharge and headache after a zip-lining trip. A combination of clinical assessment and radiological studies helped in reaching the diagnosis.

## Case presentation

A 39-year-old female with no known comorbid was in her usual state of health 10 days back when she suddenly developed nasal discharge from her right nostril after zip-lining in the northern regions of Pakistan. The nasal discharge was progressive and watery in nature. There was an associated complaint of a frontal headache, which was aggravated by bending over. There was no associated fever, neck stiffness, photophobia, or any history of trauma. The patient denied a history of any connective tissue disease, rhinitis, recurrent sneezing, or any history of previous such events.

On examination, clear fluid poured from the right nostril when the patient moved from the supine to prone position. On fundoscopy, we noted a normal cup-to-disc ratio with the absence of papilledema. The patient’s neurological examination was normal as well. Her body mass index (BMI) was calculated to be 22.

**Figure 1:**
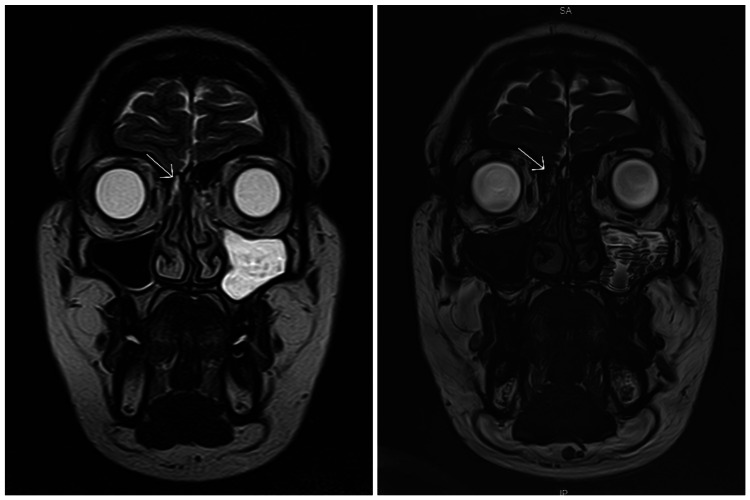
MRI (FIESTA sequence) coronal view. Asymmetric accentuated linear tract (arrow) visible in T2-weighted (left) and SSFP sequence (right) window. FIESTA, fast imaging employing steady-state acquisition; SSFP, steady-state free precession.

She was advised an MRI (fast imaging employing steady-state acquisition [FIESTA] sequence) (Figure 1), which showed an asymmetric accentuated linear tract via the right cribriform plate from the right frontal region into underlying ethmoids. The sellar and pineal regions were normal and no leptomeningeal enhancement was noted. The findings were highly concerning for CSF leak and lab confirmation was suggested. A beta-2-transferrin assay study was performed, which confirmed the diagnosis of CSF rhinorrhea.

Due to the mild nature of the patient's symptoms, she was managed conservatively with advice for strict bed rest. No prophylactic antibiotics were given. On her subsequent follow-up visit, the patient's symptoms were resolved completely.

## Discussion

CSF leak is an outcome of an abnormal communication formed between the skull base and the dura, most commonly presenting as CSF rhinorrhea. Orthostatic headache, also known as positional headache, is the telltale symptom of intracranial hypotension caused by the CSF leak [4]. Other common symptoms include rhinorrhea, neck pain or stiffness, nausea, vomiting, and hearing problems [5]. In addition to headaches, CSF leak raises the risk of meningitis and developing a brain abscess [6]. Meningitis develops in 19% of persistent CSF leak cases with a 10% mortality [7].

Spontaneous CSF leaks have traditionally accounted for only a small percentage of patients with CSF leaks, yet contemporary literature has reported a much higher number of these cases [8]. Although idiopathic, recent research reveals that spontaneous CSF rhinorrhea is due to elevated intracranial pressure and normal variations in CSF pressure, like at high altitude, coughing, and sneezing [1]. Female gender, obesity, and underlying dural abnormality are known risk factors for developing a spontaneous CSF leak [9]. An association of spontaneous CSF leaks with connective tissue disorders like Marfan syndrome has also been well-established by many studies [10].

The most common location of spontaneous CSF leak is the cribriform plate and the lateral recess of the sphenoid sinus [11]. Imaging techniques like magnetic resonance (MR) cisternography, CT cisternography, high-resolution computed tomography (HRCT) of the skull base, and radionuclide cisternography are used to detect CSF leaks. Although CT cisternography is considered the gold standard for evaluation of CSF fistula [2], our hospital did not have the facility of CT cisternography, thus an MRI FIESTA sequence was done that showed asymmetric accentuated linear tract via the right cribriform plate. MRI FIESTA sequence is sensitive, does not require an injection of intrathecal contrast, and is not associated with complications compared to CT cisternography [12].

Management of CSF leak varies as some patients are treated conservatively, others undergo endoscopic repair depending upon the severity of symptoms, risk of developing meningitis, and response to conservative management.

In our case, the patient is a female, a well-established risk factor for the condition though no other co-morbid like connective tissue disease or obesity are present. People who zip-line are most likely to get injured by any, or all of the following mechanisms, falling from the zip-line or zip-line platform, collision, trauma secondary to faulty gear, anxiety, stress, or difficulty landing [13]. In our case, the patient did not recall any trauma before, during, or after she zip-lined, but was sure that her symptoms started after she returned from her zip-lining trip. It is also essential to consider that at high altitudes (where adventure activities like zip-lining usually take place), intracranial pressure is increased [14]. This increase in intracranial pressure can also explain this episode of CSF leak in our patients.

## Conclusions

Many cases of CSF rhinorrhea after documented trauma can be found in published literature. However, our case is unique as no case associated with atraumatic zip-lining and spontaneous CSF rhinorrhea is reported. Due to the increased risk of developing meningitis, spontaneous CSF rhinorrhea is an important differential to consider when evaluating patients with persistent rhinorrhea who present with a history of zip-lining. A thorough clinical history followed by radiological evidence is essential in reaching a diagnosis of CSF rhinorrhea.
